# A comparison of two methodologies for radiotherapy treatment plan optimization and QA for clinical trials

**DOI:** 10.1002/acm2.13401

**Published:** 2021-08-25

**Authors:** Huaizhi Geng, Tawfik Giaddui, Chingyun Cheng, Haoyu Zhong, Samuel Ryu, Zhongxing Liao, Fang‐Fang Yin, Michael Gillin, Radhe Mohan, Ying Xiao

**Affiliations:** ^1^ Department of Radiation Oncology University of Pennsylvania Philadelphia Pennsylvania USA; ^2^ Stony Brook University Medical Center Stony Brook New York USA; ^3^ MD Anderson Cancer Center Houston Texas USA; ^4^ Duke University Medical Center Durham North Carolina USA

**Keywords:** knowledge based planning, PlanIQ, radiotherapy quality assurance, RapidPlan

## Abstract

**Background and purpose:**

The efficacy of clinical trials and the outcome of patient treatment are dependent on the quality assurance (QA) of radiation therapy (RT) plans. There are two widely utilized approaches that include plan optimization guidance created based on patient‐specific anatomy. This study examined these two techniques for dose‐volume histogram predictions, RT plan optimizations, and prospective QA processes, namely the knowledge‐based planning (KBP) technique and another first principle (FP) technique.

**Methods:**

This analysis included 60, 44, and 10 RT plans from three Radiation Therapy Oncology Group (RTOG) multi‐institutional trials: RTOG 0631 (Spine SRS), RTOG 1308 (NSCLC), and RTOG 0522 (H&N), respectively. Both approaches were compared in terms of dose prediction and plan optimization. The dose predictions were also compared to the original plan submitted to the trials for the QA procedure.

**Results:**

For the RTOG 0631 (Spine SRS) and RTOG 0522 (H&N) plans, the dose predictions from both techniques have correlation coefficients of >0.9. The RT plans that were re‐optimized based on the predictions from both techniques showed similar quality, with no statistically significant differences in target coverage or organ‐at‐risk sparing. The predictions of mean lung and heart doses from both methods for RTOG1308 patients, on the other hand, have a discrepancy of up to 14 Gy.

**Conclusions:**

Both methods are valuable tools for optimization guidance of RT plans for Spine SRS and Head and Neck cases, as well as for QA purposes. On the other hand, the findings suggest that KBP may be more feasible in the case of inoperable lung cancer patients who are treated with IMRT plans that have spatially unevenly distributed beam angles.

## 1 INTRODUCTION

The compliance of radiation therapy (RT) treatment plans to clinical trial protocol guidelines has been directly correlated with patient outcomes.^1^, ^2^, ^3^ RT plans submitted to trials were scored based on protocol constraints (per protocol: score 1, variation acceptable: score 2, deviation unacceptable: score 3) by the Imaging Radiation Oncology Core (IROC) of the National Clinical Trial Network for RT quality assurance (QA). The RT quality score was found to be correlated with treatment outcome.^4^ However, the strong association between RT deviations and clinical outcomes may not truly represent causation^1^; deviations from protocol guidelines may be related to unfavorable patient anatomy (e.g., tumor size, shape, and location) or the quality of the treatment plan. The purpose of QA is to identify cases that are not compliant with the protocol and illuminate the underlying reasons for non‐compliance.

A knowledge‐based planning (KBP) method that calculates achievable RT plans based on patient anatomy and past planning experience has been reported.^5^ This method was adopted and introduced as a separate module (RapidPlan) in the Eclipse treatment planning system (TPS) (Varian Medical Systems, Palo Alto, CA, USA).^6^ This module has been widely tested in clinical settings for plan optimization and QA of clinical trials.^7^, ^8^, ^9^, ^10^, ^11^, ^12^

Another method that generates direct predictions of organ‐at‐risk (OAR) dose‐volume histograms (DVHs) for treatment plans based on individual patient anatomy and dosimetry was also introduced.^13^ This method calculates the predictions based on the first principle (FP) benchmark dose with maximum dose gradients estimated around the target volume(s). PlanIQ (Sun Nuclear Corp, Melbourne, FL, USA)^13^, ^14^ is a standalone commercial platform that implements this prediction method. Unlike KBP, the FP method does not require prior knowledge or beam angles specifications. This method was tested for successful dose reduction on the contralateral parotid and larynx in clinical head and neck 4‐arc plans.^15^ PlanIQ DVH predictions have been integrated into the AutoPlan module of Pinnacle TPS (Philips Medical System, Fitchburg, WI, USA) in order to achieve more personalized prediction‐guided RT plan optimizations.^16^, ^17^ The findings showed that the integration improves OAR sparing for all disease sites by a statistically significant amount.

There has not been a comprehensive comparison of the KBP and FP methods published. Using data from multi‐center clinical trials, this study will compare these two methods for plan optimization guidance and QA for different disease sites. Instead of using Pinnacle, the FP predictions were imported into Varian Eclipse and compared to the KBP module in Eclipse for plan optimization.

## 2 MATERIALS AND METHODS

### 2.1 Materials

Randomly selected patient DICOM data submitted to the following three National Clinical Trials Network clinical trials were used in this study.

#### 2.1.1 NRG oncology RTOG 0631 (Spine SRS)

Phase II/III Study of Image‐Guided Radiosurgery/SBRT for Localized Spine Metastasis.^18^ From this trial, 50 and 10 cases were chosen at random for model construction and testing, respectively. Two of the 10 cases chosen for testing had two lesions in both the spinal cord and the cauda equina, and one case had two lesions in the spinal cord. Individual lesions were treated and assessed on their own.

#### 2.1.2 NRG oncology RTOG 1308 (NSCLC)

Phase III Randomized Trial Comparing Overall Survival after Photon versus Proton Chemoradiotherapy for Inoperable Stage II–IIIB NSCLC (only photon cases were used in this study).^19^ Thirty‐four and 10 cases were selected for model construction and testing, respectively.

#### 2.1.3 RTOG 0522 (H&N)

A Randomized Phase III Trial of Concurrent Accelerated Radiation and Cisplatin versus Concurrent Accelerated Radiation, Cisplatin, and Cetuximab (C225) for Stage III and IV Head and Neck Carcinomas.^20^ Ten cases were used for testing, and a model previously published was used for the KBP method. The dose constraints provided in the protocol were used for plan guidance and evaluations.

### 2.2 Methods

**FIGURE 1:**
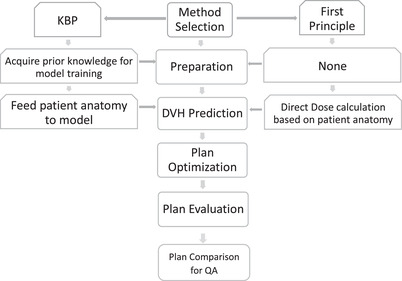
Workflow diagram depicts the process of using two methods for RT plan optimization

The flowchart depicts the workflow for data preparation, DVH prediction, plan optimization, and evaluation (Figure 1).

### 2.3 Data preparation

Prior knowledge and model construction are required by the KBP method: 50 treatment plans for patients enrolled in RTOG 0631 (Spine SRS) and 34 patients enrolled in RTOG 1308 (NSCLC) that met the contouring and dosimetric QA criteria were selected to construct the KBP models using Varian Eclipse (version 13.6.15) TPS. A previously reported Head and Neck model^21^ was explored for application on plans submitted to RTOG 0522 (H&N). The FP method, on the other hand, does not require prior knowledge to generate feasible DVH predictions.

### 2.4 DVH prediction

The CT and RT structures of testing cases were imported to Varian Eclipse TPS (v13.6.15) and PlanIQ (v2.1.1) to generate DVH predictions.

In Varian Eclipse, the line objectives of related OARs generated by the RapidPlan model were used as the feasible DVHs and plan optimizations.

Initially, the benchmark doses were calculated using PlanIQ. The prescription dose was applied to the target with a 3‐mm grid resolution and a 6‐MV dose kernel. The dose kernel was deformed based on the CT density, and a low‐dose periphery with a high gradient at the target surface was used to calculate dose spillage. High‐voltage photon‐beam dose gradients depend on numerous factors, including energy spectrum, depth in tissue, transmission of the modeling material, shape of the modulating edge, and local density of the tissue. The PlanIQ dose algorithm applies the simplest and unachievable dose gradient perpendicular to a photon beamlet (sheer gradient) with the user‐selected energy and based on the standard transmission and leaf end shape of the common Varian 120 leaf multi‐leaf collimator. The gradient also varies slightly depending on the local anatomy density based on local CT Hounsfield units.^13^

**FIGURE 2:**
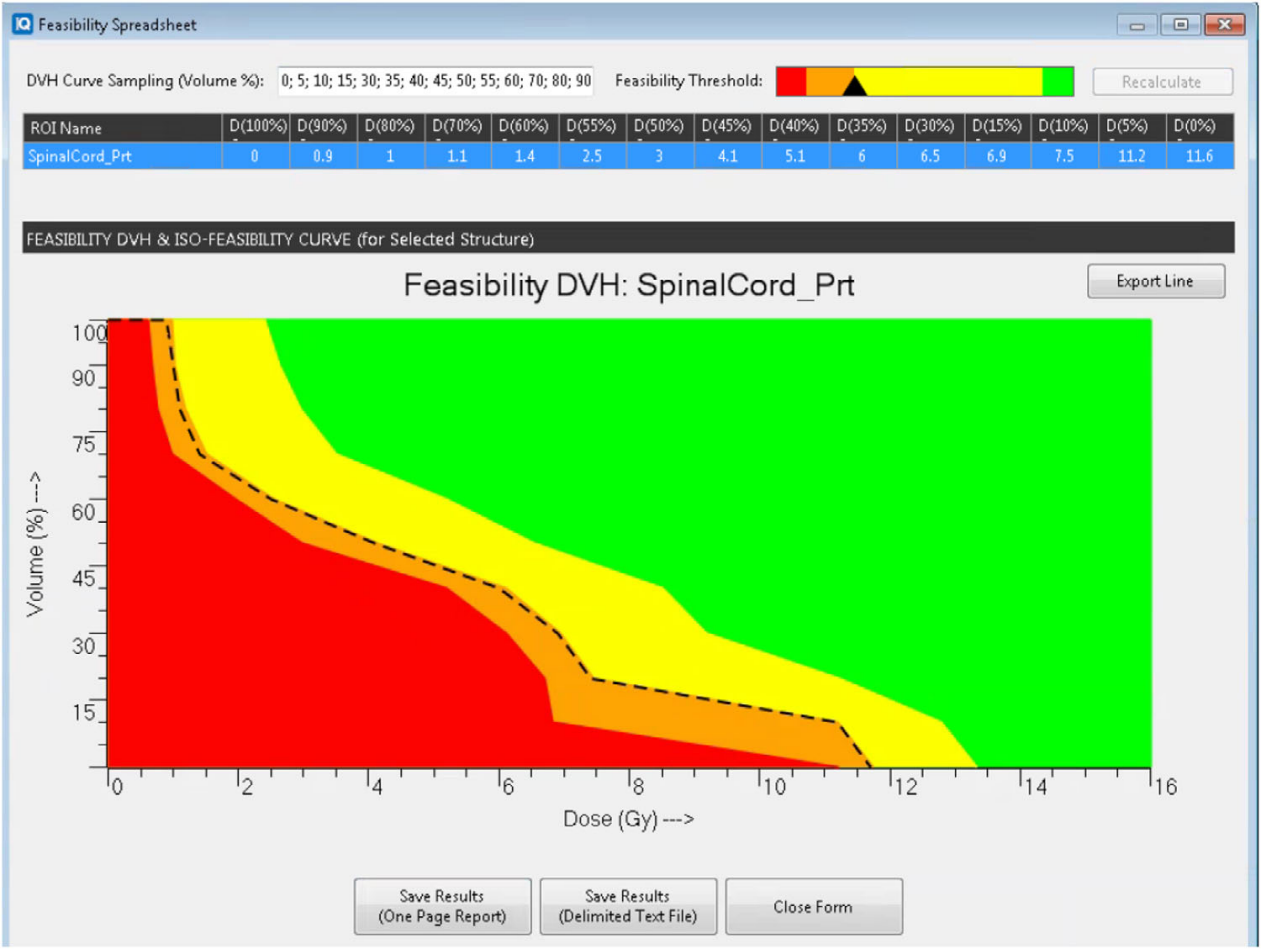
Diagram for feasible DVH estimation of SpinalCord for patient from RTOG0631

A sliding bar was provided in PlanIQ for the feasibility estimation of DVHs based on the benchmark dose, as shown in Figure 2. The estimate of achievable DVHs can be classified into the following four categories: 1. Impossible at 100% coverage (red). 2. Difficult (orange). 3. Challenging (yellow), and 4. Probable (green).^13^ The technical details of the benchmark dose and feasible DVH calculations have been previously reported.^22^

To generate DVH predictions for all prediction comparisons, the sliding bar on PlanIQ was placed between difficult and challenging (the dotted line shown in Figure 2) in this study. This sliding bar position was also used for head and neck plan optimization guidance, which aided in the generation of an RT plan with the best quality for both target coverage and OAR sparing. To meet protocol constraints, the sliding bar was shifted to the region between difficult and impossible (specifically between the red and orange range in Figure 2) to generate optimization objectives for the spinal cord for RTOG 0631 (Spine SRS). For plan optimization guidance, the DVH predictions were exported and imported into Eclipse TPS.

### 2.5 Plan optimization, evaluation, and QA

For the RTOG 0631(Spine SRS) plans, a high‐dose normal tissue ring structure was added to guarantee fast dose falloff for stereotactic radiosurgery. The ring structure is generated around the planning target volume (PTV) extending 5 mm beyond the PTV. Volumetric modulated arc therapy plans with two 360° arcs and fixed collimator angles of 0° and 90°were created for each case from RTOG 0631(Spine SRS) and RTOG 0522 (H&N). The arc geometry tool in Eclipse was used to select isocenter and jaw settings for each arc. For RTOG 1308 (NSCLC) plans, the originally submitted plan beam arrangement was used. Eclipse version 13.6 in the NRG cloud and the Photon Optimizer with a medium (2.5 mm) resolution were used. Final dose calculations were performed using the analytical anisotropic algorithm on a 2.5‐mm grid.

On each testing patient, two identical plans were generated: one using model‐generated objectives (KBP plan) and the other using FP‐predicted objectives (FP plan) for the same dosimetric parameters and priority weightings. All of the cases went through two optimization iterations.

Following that, the two plans were compared using the protocol compliance criteria listed in Tables 1 and 2. Both plans were compared to the one that was originally submitted. The targets dose conformity indices were calculated.

**TABLE 1:** Dosimetric parameter comparison of FP‐guided plan re‐optimization and KBP‐guided plan re‐optimization for 10 treatment plans submitted to RTOG 0631

RTOG 0631 Cases			FP		KBP	
Structure ID	DVH objective	Protocol constraints	Average	SD	Average	SD
PTV_1600	V16 Gy (%)	≥ 95	98%	1%	99%	1%
NonPTV1600	V16.8 Gy (cc)	≤ 2	1.13	0.78	1.32	0.71
NonPTV1600	D0.03 cc [Gy]	≤ 17.6	17.82	0.39	17.91	0.38
NonPTV1600_10	D0.03 cc [Gy]	N/A	11.32	1.01	11.35	0.96
NonPTV1600_15	D0.03 cc [Gy]	N/A	10.01	1.1	9.8	1.16
PTV_1600	D99% [Gy]	N/A	15.75	0.2	15.86	0.29
SpinalCord	D0.03 cc [Gy]	≤ 14	8.35	1.02	9.14	1.03
SpinalCord	D0.35 cc [Gy]	≤ 10	6.3	1.09	6.97	1.22
SpinalCord_Prt	D10% [Gy]	≤ 10	7.29	1.24	7.85	0.53
CaudaEquina	D0.03 cc [Gy]	≤ 16	11.96	1.01	12.05	1.43
CaudaEquina	D5 cc [Gy]	≤ 14	4.45	1.67	4.51	1.82
Esophagus	D0.03 cc [Gy]	≤ 16	10.94	5.64	11.03	5.61
Esophagus	D5 cc [Gy]	≤ 11.9	3.39	3.86	3.69	4.03
BrachialPlexus	D0.03 cc [Gy]	≤ 17.5	9.3	3.52	8.61	3.25
BrachialPlexus	D3 cc [Gy]	≤ 14	3.45	1.31	3.46	1.31
Heart	D0.03 cc [Gy]	≤ 22	6.16	4.66	6.19	4.6
Heart	D15 cc [Gy]	≤ 16	3.54	2.72	3.61	2.7
GreatVessels	D0.03 cc [Gy]	≤ 37	9.99	6.26	9.85	6.49
GreatVessels	D10 cc [Gy]	≤ 31	2.99	2.96	3.25	3.16
Trachea	D0.03 cc [Gy]	≤ 20.2	5.01	5.41	4.82	5.18
Trachea	D4 cc [Gy]	≤ 10.5	3.37	3.61	3.5	3.75
SkinOAR	D0.03 cc [Gy]	≤ 26	4.52	1.26	4.67	1.32
SkinOAR	D10 cc [Gy]	≤ 23	2.86	0.84	2.92	0.81
PTV_1600	Conformity index	N/A	85%	3%	84%	4%

**TABLE 2:** Dosimetric parameter comparison of FP‐guided plan re‐optimization and KBP‐guided plan re‐optimization for 12 treatment plans submitted to RTOG 0522

RTOG 0522 cases			FP		KBP	
Structure ID	DVH objective	Protocol constraints	Average	SD	Average	SD
PTV_7000	V65 Gy [%]	≥ 99	99.97%	0.04%	99.94%	0.16%
PTV_7000	V70 Gy [%]	≥ 95	98.04%	1.10%	97.46%	2.35%
PTV_7000	V77 Gy [%]	≤ 20	5.18%	5.90%	4.11%	4.22%
PTV_7000	V80 Gy [%]	≤ 5	0.06%	0.12%	0.05%	0.11%
PTV_7000	D95% [Gy]	≥ 70	70.84	0.41	70.7	0.72
PTV_5600	V52 Gy [%]	≥ 99	99.80%	0.24%	99.74%	0.29%
SpinalCord	D0.03 cc [Gy]	≤ 48	41.09	5.22	42.77	5.56
Larynx	D0.03 cc [Gy]	≤ 45	51.19	16.4	52.12	16.71
Parotid_Contr	Mean [Gy]	≤ 26	23.29	10.08	23.85	8.09
Parotids	Mean [Gy]	N/A	32.09	11.2	30.19	9.69
E‐PTV	V70 Gy [%]	≤ 5	0.07%	0.12%	0.03%	0.03%
PTV_7000	Conformity	N/A	91.21%	3.18%	90.50%	3.02%

For the conformity index, we used the Paddick Index as follows:

$$CI\;=\frac{TV_{PI}^{2}}{PIV\times TV}\;$$

where TV_PI_ is the target volume encompassed by the prescription isodose surface, PIV is the prescription isodose surface volume, and TV is the target volume.

The results for the originally submitted plan were compared with predictions obtained from both methods to explore the possibility of utilizing these predictions for plan QA.

## 3 RESULTS

### 3.1 Comparison of KBP and FP methods for DVH predictions

**FIGURE 3:**
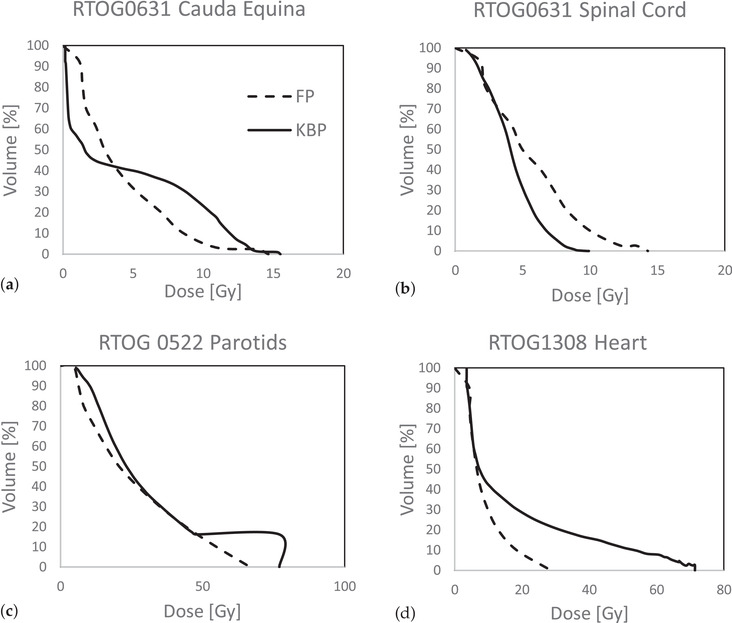
Average OAR DVH predictions comparison. For FP prediction the sliding bar was put in between challenge and difficult. (a) DVH predictions for cauda equina from RTOG 0631. (b) DVH predictions for the spinal cord from RTOG 0631. (c) DVH predictions for parotids from RTOG 0522. (d) DVH predictions for spinal cords from RTOG 1308

The average predictions of test cases in each scenario are plotted in Figure 3 using DVH predictions obtained from both the KBP and FP methods (sliding bar between challenge and difficult). To compare the two methods, specific critical OARs were used. When target coverage is not sacrificed, the high‐dose region of the KBP prediction, shown in Figure 3 RTOG 0522 (H&N) parotids, reflects the actual dose distribution on the overlapping region of parotids with target. The FP predictions for the RTOG0631 spinal cord maximum dose were around 4 Gy higher on average than the KBP predictions. The FP method calculates possible OAR doses based on uniform coverage of target with prescription dose, which is not true for SRS plans. As a result, shifting the sliding bar to a more difficult region will result in more accurate OAR dose predictions for SRS plans. The sliding bar was moved between difficult and impossible region, which was used to generate goals for RTOG0631(Spine SRS) plan optimizations to meet protocol dose constraints.

When compared to KBP prediction, the FP prediction for RTOG 1308 (NSCLC) Heart was 40 Gy lower (Figure 3). Target coverage was significantly reduced, and the dose distribution was strained as a result of plan optimization based on the FP predictions. The sliding bar position was then investigated to generate feasible dose predictions for RTOG1308 (NSCLC) heart and lungs for plan optimization guidance. The sliding bar's universal position for generating feasible predictions for all RTOG 1308 (NSCLC) test cohort patients, however, has yet to be discovered.

### 3.2 Comparison of plan re‐optimization

Tables 1 and 2 show dosimetric comparisons of the plans optimized by guidance from the KBP and FP predictions, respectively. All resulting plans met the protocol criteria.

For RTOG 0631 cases, the paired two‐tailed *t*‐test did not reveal any statistically significant differences in target coverage {V16 Gy [%] (*p* = 0.5402), D99% [Gy] (*p* = 0.2179)}, OAR sparing {SpinalCord D0.03 cc [Gy] (*p* = 0.1348), SpinalCord_Prt D10% [Gy] (*p* = 0.2788)}, high‐dose spillage to normal tissue {NonPTV V16.8 Gy [cc] (*p* = 0.3141)}, or plan conformity index for PTV_1600 (*p* = 0.1767).

For RTOG 0522 cases, the paired two‐tailed *t*‐test did not show statistically significant difference for target coverage V65 Gy [%] (*p* = 0.4876), OAR sparing (SpinalCord D0.03 cc [Gy] (*p* = 0.2938), Larynx D0.03 cc [Gy] (*p*  =  0.5211) and contralateral Parotids Mean [Gy] (*p* = 0.5139), or dose conformity index for PTV_7000.

Comparison for the RTOG 1308 (NSCLC) plan is not reported as the universal position of the sliding bar for generating feasible predictions for all RTOG 1308 (NSCLC) test cohort patients was not discovered.

### 3.3 Plan QA results

**FIGURE 4:**
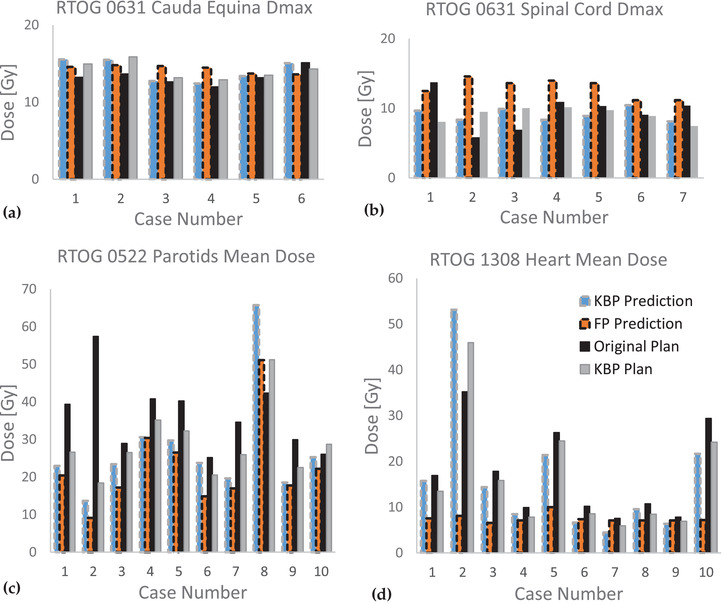
Comparison of OAR dosimetric predictions from both methods overlayed with actual plan values. (a) Cauda equina maximum dose for RTOG 0631 patients. (b) Spinal coad maximum dose for RTOG 0631 patients. (c) Parotids mean dose for RTOG 0522 patients. (d) Heart mean dose for RTOG 1308 patients

Predictions for dosimetric points of critical OARs were plotted against the original submitted plan, and the values yielded by the re‐optimized KBP plan in Figure 4.

Figure 4a,b shows the maximum dose values for the cauda equina and spinal cord, respectively, for patients in RTOG 0631(Spine SRS). The spinal cord maximum dose for original Plan 1 was 4 Gy higher than that of the KBP prediction, whereas that for Plan 2 was 2 Gy lower than that of the KBP prediction. The average prediction from the FP method (sliding bar placed in between Challenging and Difficult) was 3.8 ± 1.8 Gy (paired two‐sided *t*‐test *p* = 0.0015) higher than that of the KBP method. None of the two predictions for the cauda equina exhibited statistically significant differences (0.2 ± 1.4 Gy).

In Figure 4c, the submitted plan for case 2 yielded a parotids mean dose 48 and 43 Gy higher than the FP prediction and KBP prediction, respectively. The KBP plan reduced the parotid mean dose by 39 Gy, increased the PTV_7000 D95% [Gy] by 10.2 Gy, and reduced the spinal cord Dmax by 3.1 Gy. This indicates that both methods are useful tools for QA of parotid sparing for submitted cases. For all 10 cases, the correlation coefficient between the FP predictions and KBP predictions was 0.962, while that between the KBP predictions and re‐plan‐realized values was 0.957.

Figure 4d shows the RTOG 1308 (NSCLC) heart. The average FP prediction for the heart mean dose was 8.6 Gy lower (*p* = 0.0819) than that of the KBP prediction, 8.6 Gy lower (*p* = 0.0102) than the KBP re‐plan value, and 9.6 Gy lower (*p* = 0.0493) than the original plan‐realized value. The correlation coefficient between the KBP prediction and re‐plan‐realized value for the mean heart dose was 0.984. For individual patients treated with a few‐beam‐intensity‐modulated RT (e.g., cases 2, 5, and 10), the FP predictions deviated significantly from the achievable values.

## 4 DISCUSSIONS

The radiation oncology community in general accepts the KBP method. When compared to the FP method, it uses a more accurate dose calculation algorithm, namely the analytical anisotropic algorithm^6^ used by the Varian Eclipse TPS. Furthermore, KBP DVH predictions take into consideration the actual plan field configurations. As a result, KBP models provide more accurate predictions across the diseaes sites. However, because a plan library is required, this method is less flexible than the FP method. Because knowledge of OAR tolerable dose constraints is constantly expanding, library cases must be rebuilt to accommodate these changes.

Comparison of the FP and KBP predictions for the same cases may provide insight into library cases used in the models. In this analysis, the average spinal cord Dmax for the RTOG 0631 (Spine SRS) cases obtained from the KBP predictions was lower than that of the FP predictions (the bar placed in between Difficult and Challenging). This finding suggests that the library plans used in the RTOG 0631(Spine SRS) model sacrificed PTV coverage to meet the spinal cord dose constraint. The FP method provides flexibility to the user to generate optimization objectives for plan guidance. When the sliding bar on Figure 2 was adjusted to the red region for spinal cord DVH predictions, the realized plans exhibited statistically comparable spinal cord sparing results compared with the KBP plans.

Plan optimization for RTOG 0522 (H&N)) and RTOG0631 (Spine SRS) plans based on predictions from both methods yielded plans of similar quality, with no statistically significant differences in target coverage or OAR sparing. This indicated that the FP method provided useful predictions for plan optimization in head and neck and spine surgery cases without the need for prior knowledge.

A report^17^ on the successful use of FP predictions for planning guidance for lung cancer patients is noteworthy. Patients in the RTOG 1308 (NSCLC) study have large, irregularly shaped stage II to IIIB inoperable lung lesions. For the cohort of patients, unique angle intensity‐modulated RT techniques were used to deliver the escalated prescription dose of 70 Gy while also meeting the more stringent OAR constraints.^19^ Although the FP method was successful in generating optimization guidance in some cases, it was unsuccessful in generating attainable predictions in others.

For other disease sites not covered in this study, more comparisons and actual planning are needed. Finally, individual clinical judgment determines the applicability of FP predictions as well as the proper position of the sliding bars.

## 5 CONCLUSIONS

The KBP approach can be a reliable tool for all disease sites and clinical situations if a high‐quality plan library is available. The FP technique, on the other hand, provides quick insight into the patient's anatomy (without the need for prior knowledge) as well as flexible plan optimization guidance. However, the FP technique ignores beam geometry and relies on a less precise dose calculation algorithm. As a result, it might not be appropriate in some situations, such as individuals with inoperable lung cancers treated with a few beam IMRT in RTOG 1308.

## CONFLICT OF INTEREST

The authors declare that there is no conflict of interest that could be perceived as prejudicing the impartiality of the research reported.

## AUTHOR CONTRIBUTIONS

Huaizhi Geng and Ying Xiao conceived and designed the experiments and wrote the manuscript. Tawfik Giaddui, Chingyun Cheng, and Haoyu Zhong helped with the data analysis and paper writing. Samuel Ryu, Zhongxing Liao, Fang‐Fang Yin, Michael T Gillin, and Radhe Mohan are principal investigators and physics co‐chairs for the clinical trials related to this study.

## FUNDING INFORMATION

“This project was supported by grants U10CA180868 (NRG Oncology Operations), U10CA180822 (NRG Oncology SDMC), and U24CA180803 (IROC), from the National Cancer Institute, and in part by a grant from the Pennsylvania Department of Health. The Department specifically disclaims responsibility for any analyses, interpretations, or conclusions by Eli Lilly.”

## Supporting information

SUPPORTING INFORMATION

## Data Availability

The data that support the findings of this study are available from IROC Philadelphia RT QA. Restrictions apply to the availability of these data, which were used under license for this study. Data are available from ACR cloud service with the permission of IROC Philadelphia RT QA.

## References

[1] Ohri N , Shen X , Dicker AP , Doyle LA , Harrison AS , Showalter TN . Radiotherapy protocol deviations and clinical outcomes: a meta‐analysis of cooperative group clinical trials. J Natl Cancer Inst. 2013;105:387–393. 23468460 10.1093/jnci/djt001 PMC3601950

[2] Abrams RA , Winter KA , Regine WF , et al. Failure to adhere to protocol specified radiation therapy guidelines was associaated with decreased survival in RTOG 9704 ‐A phase III trial of adjuvant chemotherapy and chemoradiotherapy for patients with resecyed adenocarcinoma of the pancreas. Int J Radiat Oncol Biol Phys. 2012;82:809–816. 21277694 10.1016/j.ijrobp.2010.11.039 PMC3133855

[3] Fairchild A , Straube W , Laurie F , Followill D . Does quality of radiation therapy predict outcomes of multicenter cooperative group trials? A literature review. Int J Radiat Oncol Biol Phys. 2013;87:246–260. 23683829 10.1016/j.ijrobp.2013.03.036 PMC3749289

[4] Zhong H , Men K , Wang J , et al. The impact of clinical trial quality assurance on outcome in head and neck radiotherapy treatment. Front Oncol. 2019;9:792. 31497534 10.3389/fonc.2019.00792 PMC6712430

[5] Moore KL , Schmidt R , Moiseenko V , et al. Quantifying unnecessary normal tissue complication risks due to suboptimal planning: a secondary study on RTOG0126. Int J Radiat Oncol Biol Phys. 2015;92:228–235. 25847605 10.1016/j.ijrobp.2015.01.046 PMC4431941

[6] Varian Medical Systems . Eclipse Photon and Electron Reference Guide. Palo Alto, CA: Varian Medical Systems; 2014.

[7] Hussein M , South CP , Barry MA , et al. Clinical validation and benchmarking of knowledge‐based IMRT and VMAT treatment planning in pelvic anatomy. Radiother Oncol. 2016;120:473–479. 27427380 10.1016/j.radonc.2016.06.022

[8] Tol JP , Dahele M , Delaney AR , Slotman BJ , Verbakel WFAR . Can knowledge‐based DVH predictions be used for automated, individualized quality assurance of radiotherapy treatment plans?. Radiat Oncol. 2015;10:234. 26584574 10.1186/s13014-015-0542-1 PMC4653923

[9] Berry SL , Ma R , Boczkowski A , Jackson A , Zhang P , Hunt M . Evaluating inter‐campus plan consistency using a knowledge based planning model. Radiother Oncol. 2016;120(2):349–355. 27394695 10.1016/j.radonc.2016.06.010 PMC5003669

[10] Fogliata A , Wang P‐M , Belosi F , et al. Assessment of a model based optimization engine for volumetric modulated arc therapy for patients with advanced hepatocellular cancer. Radiat Oncol. 2014;9:236. 25348465 10.1186/s13014-014-0236-0 PMC4219039

[11] Fogliata A , Nicolini G , Bourgier C , et al. Performance of a knowledge‐based model for optimization of volumetric modulated arc therapy plans for single and bilateral breast irradiation. PLoS One. 2015;10:1–12. 10.1371/journal.pone.0145137 PMC4686991 26691687

[12] Giaddui T , Chen W , Yu J , et al. Establishing the feasibility of the dosimetric compliance criteria of RTOG 1308: phase III randomized trial comparing overall survival after photon versus proton radiochemotherapy for inoperable stage II‐IIIB NSCLC. Radiat Oncol. 2016;11:66. 27142674 10.1186/s13014-016-0640-8 PMC4855766

[13] Sun Nuclear Corporation . PlanIQ Reference Guide; Document 1216011, Rev C, 15 Oct 2015. Sun Nuclear Corporation, Vol. model 1216.

[14] Gintz D , Latifi K , Caudell J , et al. Initial evaluation of automated treatment planning software. J Appl Clin Med Phys. 2016;17:6167. 10.1120/jacmp.v17i3.6167 PMC5690942 27167292

[15] Fried DV , Chera BS , Das SK . Assessment of PlanIQ feasibility DVH for head and neck treatment planning. J Appl Clin Med Phys. 2017;18:245‐250. 28857470 10.1002/acm2.12165 PMC5874967

[16] Perumal B , Sundaresan HE , Ranganathan V , Ramar N , Anto GJ , Meher SR . Evaluation of plan quality improvements in PlanIQ‐guided autoplanning. Reports Pract Oncol Radiother. 2019;24:533–543. 10.1016/j.rpor.2019.08.003 PMC6796777 31641339

[17] Xia W , Han F , Chen J , Miao J , Dai J . Personalized setting of plan parameters using feasibility dose volume histogram for auto‐planning in Pinnacle system. J Appl Clin Med Phys. 2020;21:119–127. 32363757 10.1002/acm2.12897 PMC7386185

[18] Ryu S , Hospital HF , Co‐chair N , et al. PHASE II /III Study of Image‐Guided Radiosurgery / SBRT for Localized Spine Metastasis. NRG Oncology. 2011.

[19] Liao ZX , Bradly J , Gillin M , Mohan R . Phase III Randomized Trial Comparing Overall Survival after Photon Versus Proton Chemoradiotherapy for Inoperable Stage II‐IIIB NSCLC. NRG Oncology. 2013;2013:1–84.

[20] Axelrod R , Ang K , Rosenthal M . Rtog 0522: a randomized phase III trial of concurrent accelerated radiation and cisplatin versus concurrent accelerated radiation, cisplatin, and cetuximab (C225) [followed by surgery for selected patients] for stage III and IV head and neck carcinom. 2009 17344795

[21] Giaddui T , Geng H , Chen Q , et al. Offline quality assurance for intensity modulated radiation therapy treatment plans for NRG‐HN001 head and neck clinical trial using knowledge‐based planning. Adv Radiat Oncol. 2020;5(6):1342–1349. 33305097 10.1016/j.adro.2020.05.005 PMC7718499

[22] Ahmed S , Nelms B , Gintz D , et al. A method for a priori estimation of best feasible DVH for organs‐at‐risk: validation for head and neck VMAT planning. Med Phys. 2017;44:5486–5497. 28777469 10.1002/mp.12500

