# Supplementary material for: A comparison of two methodologies for radiotherapy treatment plan optimization and QA for clinical trials
Source: J Appl Clin Med Phys. 2021 Aug 25;22(10):329–37. doi: 10.1002/acm2.13401 (PMC8504592; doi:10.1002/acm2.13401)
Supplement: Supplementary file 1 — SUPPORTING INFORMATION [file ACM2-22-329-s001.docx]

A Comparison of Two Methodologies for Radiotherapy Treatment Plan Optimization and QA for Clinical Trials

Huaizhi Geng, Ph.D., University of Pennsylvania, hgeng@sas.upenn.edu

Tawfik Giaddui, Ph.D., University of Pennsylvania [tgiaddui@gmail.com](mailto:tgiaddui@gmail.com)

Chingyun Cheng, Ph.D., University of Pennsylvania, chingyun.cy.cheng@gmail.com

Haoyu Zhong, Ph.D., University Pennsylvania,  [haoyu_zhong@126.com](mailto:haoyu_zhong@126.com)

Samuel Ryu, Stony Brook University Medical Center sryu@stonybrookmedicine.edu

Zhongxing Liao, MD, MD Anderson Cancer Center, [zliao@mdanderson.org](mailto:zliao@mdanderson.org)

Fang-Fang Yin, Duke University Medical Center, fangfang.yin@duke.edu

Michael Gillin, Ph.D. MD Anderson Cancer Center,  [MGillin@mdanderson.org](mailto:%20MGillin@mdanderson.org)

Radhe Mohan, Ph.D. MD Anderson Cancer Center, [rmohan@mdanderson.org](mailto:rmohan@mdanderson.org)

Ying Xiao, Ph.D. University of Pennsylvania, [Ying.Xiao@pennmedicine.upenn.edu](mailto:Ying.Xiao@pennmedicine.upenn.edu)

**Keywords: PlanIQ, RapidPlan, Knowledge based planning, Radiotherapy Quality Assurance**

**Acknowledgments:**

**Author Contributions:** Huaizhi Geng and Ying Xiao conceived and designed the experiments and wrote the manuscript; Tawfik Giaddui, Chingyun Cheng and Haoyu Zhong helped with the data analysis and paper writing; Samuel Ryu, Zhongxing Liao, Fang-Fang Yin, Michael T Gillin, and Radhe Mohan are Principal Investigators and physics co-chairs for the clinical trials related to this study.

**Funding sources:** “This project was supported by grants U10CA180868 (NRG Oncology Operations), U10CA180822 (NRG Oncology SDMC), and U24CA180803 (IROC), from the National Cancer Institute, and in part by a grant from the Pennsylvania Department of Health. The Department specifically disclaims responsibility for any analyses, interpretations, or conclusions by Eli Lilly.”

Corresponding author: Huaizhi Geng, Ph.D, Dept. of Radiation Oncology, University of Pennsylvania, [hgeng@sas.upenn.edu](mailto:hgeng@sas.upenn.edu) ([huaizhi.geng@pennmedicine.upenn.edu](mailto:huaizhi.geng@pennmedicine.upenn.edu))

## Data Availability: Data subject to third party restrictions:

## The data that support the findings of this study are available from IROC Philadelphia RT QA. Restrictions apply to the availability of these data, which were used under license for this study. Data are available from ACR cloud service with the permission of IROC Philadelphia RT QA.
